# Promoting Scientific Transparency to Facilitate the Safe and Open International Exchange of Biological Materials and Electronic Data

**DOI:** 10.3390/tropicalmed2040057

**Published:** 2017-10-31

**Authors:** Kenneth B. Yeh, Corina Monagin, Jacqueline Fletcher

**Affiliations:** 1 MRIGlobal, Gaithersburg, MD 20878, USA; 2 One Health Institute, School of Veterinary Medicine, University of California, Davis, CA 95616, USA; cgmonagin@ucdavis.edu; 3 National Institute for Microbial Forensics & Food and Agricultural Biosecurity, Oklahoma State University, Stillwater, OK 74078, USA; jacqueline.fletcher@okstate.edu

**Keywords:** biosafety and biosecurity, emerging pandemic threats, material data transfer, one health, public health preparedness, scientific transparency

## Abstract

Scientific communication, collaboration and progress are enhanced through the exchange of data, materials and ideas. Recent advances in technology, commercial proprietary discovery and current local and global events (e.g., emerging human, animal and plant disease outbreaks) have increased the demand, and shortened optimal timelines for material and data exchange, both domestically and internationally. Specific circumstances in each case, such as the type of material being transferred (i.e., select agent, disease-causing agent and assessed biosafety risk level) and current events, dictate the level of agreements and requirements. Recent lessons learned from emerging disease issues and emergencies have demonstrated that human engagement and increased science diplomacy are needed to reinforce and sustain biosafety and biosecurity practices and processes, for better scientific transparency. A reasonable and accepted framework of guidance for open sharing of data and materials is needed that can be applied on multiple cooperative levels, including global and national. Although numerous agreement variations already exist for the exchange of materials and data, regulations to guide the development of both the language and implementation of such agreements are limited. Without such regulations, scientific exchange is often restricted, limiting opportunities for international capacity building, collaboration and cooperation. In this article, we present and discuss several international case histories that illustrate the complex nature of scientific exchange. Recommendations are made for a dual bottom-up and top-down approach that includes all stakeholders from beginning negotiation stages to emphasize trust and cooperation. The broader aim of this approach is to increase international scientific transparency and trust in a safe and open manner, supporting increased global one health security.

*Clarity and transparency are important. The kinds of things many of us are doing can help in improving people’s lives. But it is not always clear that it is a good idea to label it “diplomacy”*. John Boright, Executive Director, International Affairs, U.S. National Academies of Science, Engineering and Medicine

## 1. Introduction

Transparency in science has been defined as ‘the outcome of a suite of behaviors which characterize reproducible research’; it ‘facilitates and enhances research quality, research integrity and trust’. Transparency and openness have long been recognized norms of professional behavior in the conduct and reporting of scientific research [1,2,3,4]. Scientists formulate hypotheses and access resources to pursue further research in a process that builds successively through time and across laboratories, institutions and even national boundaries. Transparency underpins the process by which peer scientists are able to reproduce and independently validate results of previous research. In practice, it is an expression of the scientists’ core ethical and professional values such as honesty, responsibility and trust. The need for transparency applies not only to research approaches and technologies, but also to the sources and validations of any biological materials (e.g., cell lines, clinical specimens, environmental samples and nucleic acids) and/or electronic data (e.g., sequences and code) [1] on which the investigations are based. Specific and open acknowledgment of any sharing of biological materials and data among collaborating research groups provides appropriate recognition, as well as enhancing the transparency and trust that are critical as research projects become more multi-disciplinary and complex [5].

Recent trends, however, have strayed somewhat from the traditional customs of openness and sharing in scientific endeavor. While scientists conducting research in industry settings have always been more restricted in what information and materials could be shared, legal constraints, the pressures of increasing competition for grant funds, and the demands of tenure and promotion, have led some academic scientists to similar reluctance to detail specifics and to exchange materials without restriction [1,2,4]. In addition to biosafety and biosecurity (BS&S) issues, openness and sharing among scientists throughout the global community of science may be hampered by additional factors including political tensions and questions of national sovereignty or the protection of national treasures such as germplasm or breeding stock. 

Challenges to the exchange of biological materials and data include BS&S implications, the financial status of a respective country and the elements and dynamics of human engagement. Biological agents are classified according to laboratory biosafety (risk) and biodefense-related assessments [6]. For example, the classification of Ebola virus (EBOV) and other related filoviruses that cause hemorrhagic fevers may differ depending on which major organizational or national biological agent list is in use at that location (most lists have four categories). The European Union directive No. 2000/54/EC [7], World Health Organization laboratory biosafety manual [8] and US National Institutes of Health guidelines for research involving recombinant molecules [9], classify EBOV under risk group 4, consisting of the most dangerous agents carrying the highest risk. In contrast, the Chinese classification and the Russian national sanitary regulations (SR) rank the levels in reverse numerical order such that risk group 1 and pathogenicity group I, respectively, which include EBOV and Marburg viruses, are the most dangerous [10,11]. While a comparison of major biological agent lists is consistent among risk groups, these discrepancies and multiple listings highlight the importance of communication and coordination of information, especially related to agreement on standard operating procedures for countries lacking formal biosafety regulations during a pandemic response.

In the United States (US), for example, certain types of materials are harder than others to exchange due to the Centers for Disease Control and Prevention’s (CDC) Federal Select Agent Program (FSAP) regulations and policies. Other sensitive items include sovereign materials designated as ‘national treasures’ (e.g., native animal, plant species or similar) and any materials and data considered intellectual property (IP). Such exchanges will be subject to regulations of the US CDC, Department of Health and Human Services (HHS), Food and Drug Administration (FDA) and Environmental Protection Agency (EPA) BS&S [12] and to FSAP-USDA permissions for possessing, using and transferring select agent materials. They will be guided also by both national/local laws and practices for respective countries since, like the US, the majority of nations across the globe restrict the types of materials that can be moved outside of national borders. For optimal international research cooperation, it is important for both parties to recognize each other’s respective regulations and policies.

**Figure 1:**
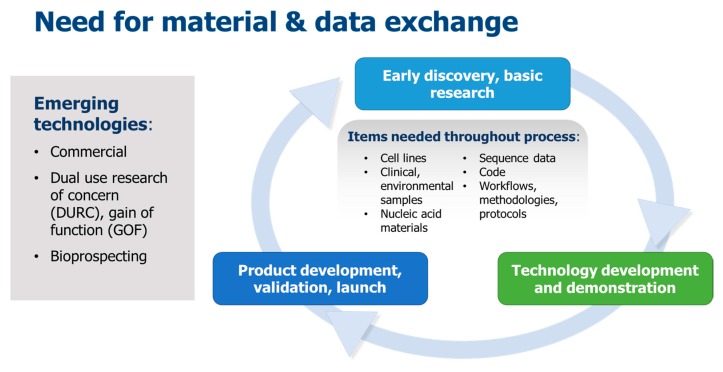
Typical biotechnology research and development process. Material and data exchange is required especially for emerging technologies.

While there is an increased need and demand for the exchange of biological materials and data, recent high-impact events have resulted in shortened timelines for effective interchange of such resources. Factors such as intensified international interactions including trade and travel, as well as new and emerging human, animal and plant diseases, unprecedented movement of refugees and climate change have increased the urgency for response and recovery activities. Such considerations are especially relevant with respect to emerging biotechnological applications such as those qualifying as dual-use research of concern (DURC) and gain-of-function (GOF) research, in which the materials and data are needed throughout a typical research and development process (Figure 1). Certain biological materials, data and technological advances, often generated during basic research, may be needed also in later phases as that technology develops into a mature product. A 2017 Global Trends report summarized this trend: ‘technological innovation accelerates progress but leads to discontinuities’ [13]. On a wider scale, this trend also factors into the analysis threats such as the likelihood of a future disease outbreak [14].

The financial status of a country, as designated by the World Bank based on gross national income per capita, can also influence the ease of scientific openness and exchange. For example, there may be greater reluctance on the part of low-income and low-middle income governments to share what may be perceived as proprietary or national assets with those of high-income countries for fear that these assets may leave the country without any gain (scientific or otherwise) to the local government. Tensions may occur between program recipients and funders, the latter of which are often from high-income countries, especially when program objectives are not previously agreed upon and expectations have not been spelled out. For example, during the 2014–2015 West Africa EBOV outbreak, tensions occurred between those local and international groups providing medical treatment (for EBOV and other diseases) and those conducting clinical trials and research studies [15]. The tensions in a collaboration may be exacerbated if one partner country lacks the experience and resources necessary to exchange materials and data, and the other partner country does not fully recognize or understand these gaps.

## 2. Case Histories Illustrating Issues Related to International Scientific Exchange of Materials and Data

To further explore these challenges to transparency and trust we describe a set of three actual case histories, one of which involved a conventional exchange of material and data, and two in which lessons learned underscored the importance for science diplomacy to engage partners, build trust and derive strategies to facilitate exchange of materials and data with the proper BS&S assurances. 

### 2.1. Case History 1

In a typical research collaboration, a material transfer agreement (MTA), often developed among the collaborators at the working level, defines exactly what material and data will be exchanged and used, who are the provider and recipient, and by what means the exchange will take place. This agreement also documents any restrictions on the use of the materials and data received during and after the specific collaborative project. Examples of straightforward material and data exchanges include a user accessing strain material from an established culture collection and a user uploading sequence data to GenBank. The MTA often supports a specific project agreement that defines the scope and period of performance for the respective collaboration, while a memorandum of understanding (MOU) establishes an agreement. MTAs and project agreements, but not MOUs, (in most cases) are legally binding contracts. Additional national level documents may include a formal umbrella agreement establishing intent between two governments and an implementing agreement specific to a given government agency. Assuming that two countries have formal diplomatic relations, national level agreements may be established as needed, as in the case of one government funding a program for another. Areas that may be covered by national and working level agreements are mostly undefined and often are developed on a case-by-case basis. 

Case history 1 involved a US Government (USG)-funded research and capacity-building program operating in multiple African and Asian countries. This program was implemented by a US institution that collaborated with national governments and local institutions within participating partner countries. In this example, we focus on material exchanges between the US institution (provider) and a recipient partner country in Asia. The US institution initiated a collaboration with a national government laboratory in the partner country, and knowledge (protocols) and reagents were passed from provider to recipient to increase the capacity of research in the partner country (and, with local dissemination, the Asian region). An MTA between the cooperating institutions in each country defined the conditions of the exchanges. In addition, the partners signed agreements regarding publication, authorship and ownership of the materials and research products. Because the program was funded by the USG there were also higher level, government-to-government national umbrella agreements that allowed the program to operate within the partner countries borders.

In our example, the cooperation resulted in the discovery of a previously-unknown animal pathogen. Due to the BS&S implications of the limited capability and resources available in the partner country, both partners decided to send materials out of the partner countries for additional analysis in the US. Additional MTAs specific to the sharing of the new materials were signed by the partner institutions, while the original documents regarding publication and authorship, signed previously, still remained valid. 

This is an interesting example of bilateral exchanges of materials from provider to recipient, and from recipient back to provider, resulting in a novel discovery as well as capacity building. The initial agreements allowed the first exchange of materials from the US to the recipient partner country and later agreements allowed for the shipment of materials out of the Asian country. National-level agreements already in place allowed for the larger program to operate in country, but the specifics of the technology/material transfer were left to the program, allowing the partners to work out appropriate, relevant mechanisms of exchange. This structure facilitated an increase of capacity in scientific exchange and international cooperation for the partner country, promoting trust and transparency.

### 2.2. Case History 2

Exchanging biological strain material, such as organisms or tissue samples is beneficial for testing diagnostic assays and allows for the generation of additional material for further validation. Case history 2 involves a recent USG-funded cooperative research project with partner country scientists in a former Soviet Union (FSU) republic to investigate archived capripox viruses (goat pox and sheep pox), which are important livestock pathogens and USDA select agents. Under already-existing umbrella and implementing agreements between the US and this partner country there was an expectation for the exchange of data and material, not only to demonstrate scientific transparency but to enhance the quality of research and raise the visibility of the partner scientists. However, the partner country had reservations regarding exchanging materials, especially those that would be exported outside their country. Besides the BS&S issues related to importing and exporting material (i.e., nucleic acid samples) there was also a perception that this transfer could result in potential attribution (i.e., in a hypothetical scenario where an infectious disease event might be traced back to their strain as a source) and/or a loss of control, use and ownership of their material and data (e.g., a hypothetical commercial scenario in which a foreign partner claims scientific credit and/or profit) [16]. In this work, strict national export control in the partner country prevented the exchange of strain material. In addition to these issues, limited political willpower hampered the ability of administrators to make important decisions related to exchanging material [17]. 

Further negotiation among the US and partner country scientists and the program funder led to a compromise that provided for exchanging electronic sequencing data only, with project agreements detailed in a standard MTA. During the project partner country scientists shared electronic sequencing data files with US collaborators via open source file sharing and storage, which proved to be simple and effective. This initial exchange of sequencing data, which facilitated the success of several collaborative studies, was a positive step leading to continued trust and further transparency by encouraging the potential for further exchange of materials.

### 2.3. Case History 3

Case history 3 consisted of international efforts during the recent EBOV outbreak in the West African countries of Guinea, Sierra Leone and Liberia. This outbreak was the largest since the virus was first identified in the Democratic Republic of Congo in 1976 [18]. In ten countries, although approaches and regulations for biosafety in research laboratories existed at the national level for EBOV, the available information varied and international norms have yet to be developed [19]. A large influx of international aid supported clinical care, epidemiological case tracking, data management, laboratory diagnosis, and research. In addition to declaring the outbreak a public health emergency, and due to the scale of the outbreak and limited knowledge of the virus itself, the World Health Organization (WHO) deemed that there was an ethical and public health obligation for research to be a part of response efforts [20]. Outcomes of researchers’ efforts, while significant, were unfortunately not as extensive as they could have been. Multiple factors, including limited in-country health and research infrastructure and capacity, logistical and social challenges such as fear and mistrust, and limited bureaucratic experience in dealing with large numbers of international partners overwhelmed ethical review boards and emergency operating centers. Further, inadequate or non-existent capacity for data and material transfer hampered the outcomes [21,22]. 

Due to limited in-country capacity in low and low-middle income countries, the exportation of material and data is a complex process having undefined timelines. During the EBOV outbreak, the many biological samples generated within the three affected countries constituted a health risk. In most instances, due in part to BS&S concerns related to the limited local capacity for safe sample storage and analysis, such samples were either exported out of those countries or destroyed. Because of the emergency conditions, some were moved without proper permits or permissions. Those having access to these exported samples included international partners involved with the response and collaborators from international academic institutions and commercial organizations [23]. Although case history 3 involved human biological samples, similar considerations would apply also to other types of materials or data.

Regulatory systems in place within the West African region to support sample transfer at the time of the EBOV outbreak were slow and cumbersome, and often consisted of processes that were not clearly defined or capably overseen. Furthermore, the crisis mode, lack of regulation and need for an urgent and immediate response resulted in a situation in which there was little accountability [23]. Ethical review boards and legislative bodies responsible for reviewing protocols and sample transfer documents were overwhelmed with the sheer amount of materials to review, and had limited training and capacity to manage such an event. As a result, research was often delayed and regulations were often bypassed [24]. 

While some ethical committees and legislative bodies had safeguards in place to ensure that MTAs accompanied sample transfer, there is testimony suggesting that not all partners followed such regulations, due to the slow nature and complexity of the process [20]. Studies estimate that approximately 80,000 biological specimens existed after the outbreak, although their precise locations have not been fully reported [25,26]. Aside from the BS&S risks resulting from improperly-shipped infectious sample material, the lack of process and standard operating procedures (SOP) for sample records and duplicate aliquots resulted in potential loss of vital sample data integrity. There was an overall need for training to fill the gaps created by lack of knowledge and experience in sample transport and sharing. Disregard for national government regulations and international scientific standards, such as the use of MTAs seen in this case example, increases mistrust within the international research community and hinders local research capacity building [27]. Repercussions of such events in the case of the West African EBOV outbreak led to:

- Rapid development and mismanagement of legislative review bodies that had little experience or capacity to deal with contracts and material transfer documents.
- Mistrust of the international aid and research community and decreased transparency between partner nations.
- Loss of ability to track data and materials exported overseas and incomplete sample sets.
- Limited ability to link associated metadata with samples that were exported or destroyed.
- Tensions between funders/donors and recipients [21,22,25,26,27].

Examples in the literature point to research difficulties due to competition between research teams [28] and overwhelmed ethical committees [22], primarily in the case of clinical trials [21]. The principle expressed in these examples from the literature can be applied easily to other scenarios of research and data transfer in an emergency. We agree with recommendations that low- and low-middle income countries must improve their capacity for legislation and rapid ethics reviews to support MTAs and data sharing [21,29] and that these discussions must be further encouraged on international and national levels to focus on specific legislations that address authority, movement and biosafety and biosecurity of samples [23]. 

The EBOV outbreak in West Africa is a poignant example of how emerging infectious diseases are risks that affect the global community. While the international community prioritized research and aid in this example, scientific transparency and trust may have been a casualty of over-stressed or inadequate local infrastructure and capacity. There must be much more emphasis on support of all partners involved to improve and invest in capacity building, specifically targeting improved scientific transparency. Researchers operating in international environments have an obligation, even during emergency events, to advocate for further scientific diplomacy and political facilitation of data and material exchange [21].

The case histories presented here focus on research pertaining specifically to human and animal pathogens but many of the same challenges are also present in international research co-operations in which the exchange of material, samples or data related to non-human organisms is desirable (Table 1). Such work may pertain specifically to plants and animals (including insects and invertebrates) from agricultural or environmental settings and matrices, and the microbes that inhabit their microbiomes in beneficial, neutral or antagonistic relationships. These biological elements are central to investigations related to disease epidemiology or management, agricultural productivity or genetic improvement, climate change or pollution impacts, microbial evolution and food safety and security, all of which are intimately intertwined with the investigation of biological materials and data. When these areas are the subject of collaborative international research projects, those involved with the planning and execution of research activity and sample exchange are obliged to follow the same, or very similar, policies and regulatory oversight. 

## 3. Conclusions and Recommendations

While laboratory biosafety is a local concern with scientists at their own workplaces, international scientific collaboration often requires minimum common levels of biosecurity practice and regulatory oversight [30]. Likewise, the synthesis of ideas, research, material exchange and data and scientific transparency as a whole begins at the local level. Our recommendations to increase scientific exchange with transparency and trust include the following:

- For the future, we suggest that individual international research collaborations develop, at the local research level, a dual bottom-up, top-down working plan that allows for multi-sectoral engagement of higher-level stakeholders (program funders, policy makers and commerce, diplomatic and trade representatives) as appropriate to that program.
- Any existing procedures and requirements, especially permissions and permits required by the proposed international cooperation, should be thoroughly investigated before proposal submission.
- When possible, collaborative agreements should reference and build upon existing tools such as the Nagoya protocol, the global health security agenda (GHSA), IHR and WHO pandemic influenza preparedness (WHO PIP), particularly when dealing with issues of biosecurity and biosafety.
- We suggest building capacity of all countries, particularly those of low- and middle-income, to establish processes to assist with scientific transparency and material and data sharing.

**Figure 2:**
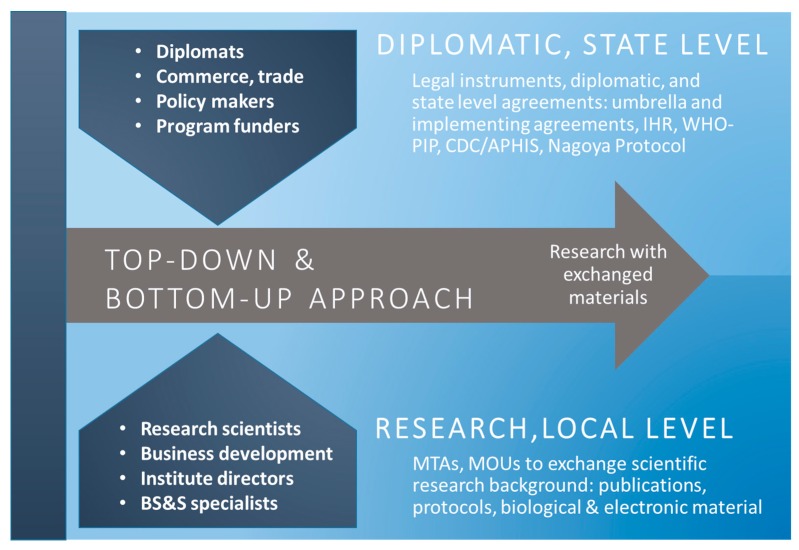
Dual top-down and bottom-up approach to yield research with exchanged materials and data. Parallel approach with diplomatic, state, and research level stakeholders is needed to better enhance research and scientific transparency.

The dual bottom-up, top-down working plan should reflect both the research study objectives and specific expected outputs in a parallel and stepwise approach (Figure 2). To better exact expectations, the history and examples of material and data exchanged including challenges (i.e., What were the obstacles? Was the timeline met?), risks and mitigations should be openly discussed by researchers, program funders and policymakers as early in the collaborative process as possible. All research outcomes should feed back into the research work flow and the working plan should be an iterative process for the duration of the collaboration to account for new developments and needs. For example, one early biosecurity question is what material is being exchanged and where is it currently being stored? A later biosecurity question might be how to ship newly identified materials across internationals boundaries?

Regarding existing agreements, for example, if an international partner country already has a strategic plan at either a national (multi-sectoral) or institute level, that plan can be adapted for application to a new proposal by supplementing with procedures and additional guidance from other existing frameworks such as the Nagoya protocol on access and benefit sharing (ABS) [31] and WHO PIP [32], both of which include tools and model documents (e.g., MTAs) for sharing materials and data. The Nagoya protocol of 2014, which aims to provide a legal guidance for providers and users of genetic material, is supplementary to the international convention of biological diversity agreement of 1993. Establishing consistent conditions will ensure that, when a research collaboration yields overall conservation and biodiversity enhancement, participating countries that provide genetic resources will share in the benefits [31]. The WHO also considers the PIP framework an access and benefit sharing instrument; however, it is specific to sharing of influenza viruses and related sequences [32]. 

BS&S considerations are critical elements of any international research collaboration and several existing tools can and should be used as references when developing agreements and planning scientific exchange of biological materials and data. Capacities and objectives for BS&S are included in the global health security agenda (GHSA) [33] and its predecessor, the international health regulations (IHR) [34]. The GHSA is a framework of 44 participating nations that further promotes IHR (2005) core capacities through peer-mentorship among countries. The GHSA’s nine objectives are presented across a framework of three foci: detect, prevent and respond. The IHR is a legal instrument binding the 194 WHO members to self-report and to assess detection and response to disease outbreaks [33]. However, political, economic, technical and operational challenges have hampered effective implementation of IHR core capacities, especially in resource-constrained areas [35]. The US CDC/NIH manual on biosafety in microbiological and biomedical laboratories (BMBL) (2009) provides internationally accepted BS&S guidelines that incorporate NIH and WHO risk group classifications. The Australia group (2008) provides guidance for export control on biological material and dual-use items.

Both the Nagoya protocol, which is recognized by state signatories, grants sovereignty of genetic resources to those states [36] and the WHO PIP include requirements to share biological materials [37] and recommend the sharing of sequencing data [32]. However, the implementation of the material and/or data exchanges will be subject to local and national laws of the countries involved. These requirements further reinforce the value of a dual bottom-up, top-down approach. Long-term stability in the cooperation will be facilitated by the creation of a back-up to the working plan, providing for defined alternatives (e.g., exchanging electronic sequencing data in cases where movement of biological samples is prohibited) and addressing emerging technologies such as epidemiological computer modeling that may include metadata and attribution sources. In addition, as we saw with the EBOV outbreak, nations should be proactive in establishing processes to assist with scientific transparency and material and data sharing so that there is guidance in place if and when an emergency arises. 

The concept of ‘science for diplomacy’ is widely recognized and practiced among the global community to enhance mutual science cooperation and build closer relationships between countries [38], however, there is less focus on ‘diplomacy for science’, a top-down approach that uses diplomatic resources to facilitate scientific cooperation [39]. But diplomacy for science does not meet all the needs of international scientific research co-operations either. Rather, since it often involves major complex aims and policies, it engages only top-level stakeholders and can neither be assumed to exist nor solely relied upon. Researchers and scientists need transparency from the bottom up as well they need access to diplomatic resources including heads of local research institutions, embassy staff who are familiar with local knowledge and customs for that respective country, commerce and export control experts.

Understanding that there is no universal model to enable and promote exchange of material and data, the current GHSA model also proposes a task-specific committee to facilitate an assessment approach that is both bottom-up and top-down. Promoting BS&S systems is also a primary objective. GHSA-recommended activities include country self-assessments via joint external evaluation (JEE), in which one country assists another through working level mentorships that teach current global health security to next generation professionals. Similarly, related topics such as new technology, especially gain of function or dual use research of concern, which have BS&S concerns but are not part of GHSA, could be addressed. Inculcating an organizational culture of BS&S systems and responsible conduct of research at the local level would further reinforce activities, especially when international organizations and professional societies that further promote international norms are engaged [40].

While developing a working plan that details the exchange of material and data for a research study may seem straightforward, we describe the importance of human engagement during this process. Lessons learned from the case histories related here confirm that successful exchanges of material and data depend on promoting, developing and earning trust among parties (providers and recipients). The trust factor in human engagement at both the political (diplomatic) and working (scientific research) levels are key to promoting scientific transparency for exchanging materials and data, especially in the areas of capacity building and cooperative research, and for the enhancement of biosafety and biosecurity.

## Figures and Tables

**Table 1:** Summary of Case Histories.

Case, Location	Umbrella Agreement	Implementing Agreement	Material Transfer Agreement	Exchanged Outputs	Issues	Outcomes
1. (Asia)	Yes	Yes	Yes	Reagents, reference material, and protocols. PCR product.	High level national agreements did not specify MTA.	Bilateral exchange of material led to discovery of a novel pathogen.
2. (Former Soviet Union)	Yes	Yes	Yes	Sequencing data only.	Strict export control prevented material exchange.	Study completed with exchanged sequencing data in lieu of sample materials.
3. (Africa; Ebola virus; EBOV)	No	No	Yes	Positive Ebola samples shipped without proper permits.	Lack of sample record keeping and fidelity with missed research opportunities and bypassed regulations.	Development of legislation supporting capacity building, increasing scientific exchange and research.
